# Comparing regression modeling strategies for predicting hometime

**DOI:** 10.1186/s12874-021-01331-9

**Published:** 2021-07-07

**Authors:** Jessalyn K. Holodinsky, Amy Y. X. Yu, Moira K. Kapral, Peter C. Austin

**Affiliations:** 1 Department of Clinical Neurosciences, Cumming School of Medicine, University of Calgary, 3330 Hospital Drive NW, Calgary, AB T2N4N1 Canada; 2 ICES, Toronto, ON Canada; 3 Department of Medicine (Neurology), University of Toronto, Sunnybrook Health Sciences Centre, Toronto, ON Canada; 4 Department of Medicine (General Internal Medicine), University of Toronto and University Health Network, Toronto, ON Canada; 5 Management, and Evaluation, Institute of Health Policy, University of Toronto, Toronto, ON Canada; 6 Schulich Heart Research Program, Sunnybrook Research Institute, Toronto, ON Canada

## Abstract

**Background:**

Hometime, the total number of days a person is living in the community (not in a healthcare institution) in a defined period of time after a hospitalization, is a patient-centred outcome metric increasingly used in healthcare research. Hometime exhibits several properties which make its statistical analysis difficult: it has a highly non-normal distribution, excess zeros, and is bounded by both a lower and upper limit. The optimal methodology for the analysis of hometime is currently unknown.

**Methods:**

Using administrative data we identified adult patients diagnosed with stroke between April 1, 2010 and December 31, 2017 in Ontario, Canada. 90-day hometime and clinically relevant covariates were determined through administrative data linkage. Fifteen different statistical and machine learning models were fit to the data using a derivation sample. The models’ predictive accuracy and bias were assessed using an independent validation sample.

**Results:**

Seventy-five thousand four hundred seventy-five patients were identified (divided into a derivation set of 49,402 and a test set of 26,073). In general, the machine learning models had lower root mean square error and mean absolute error than the statistical models. However, some statistical models resulted in lower (or equal) bias than the machine learning models. Most of the machine learning models constrained predicted values between the minimum and maximum observable hometime values but this was not the case for the statistical models. The machine learning models also allowed for the display of complex non-linear interactions between covariates and hometime. No model captured the non-normal bucket shaped hometime distribution.

**Conclusions:**

Overall, no model clearly outperformed the others. However, it was evident that machine learning methods performed better than traditional statistical methods. Among the machine learning methods, generalized boosting machines using the Poisson distribution as well as random forests regression were the best performing. No model was able to capture the bucket shaped hometime distribution and future research on factors which are associated with extreme values of hometime that are not available in administrative data is warranted.

**Supplementary Information:**

The online version contains supplementary material available at 10.1186/s12874-021-01331-9.

## Background

Hometime, defined as the total number of days a person is living in the community (not in a healthcare institution) in a defined time period after a hospitalization for a health condition, is a patient outcome metric increasingly being used in heart failure, atrial fibrillation, surgical, and stroke research [1–11]. Hometime has also been referred to as “days alive and out of hospital” and can be calculated across any time period of clinical relevance; commonly used timeframes for hometime calculation are 30, 90, 180, and 365 days. This metric has several advantages in clinical research. First, hometime be obtained using linked administrative data, making it more resistant to loss to follow up/attrition bias and it can be calculated for large populations. Second, unlike clinical outcome scores which may be vulnerable to low inter-rater reliability, hometime is an objective measure of outcome. Finally, this metric is valued by patients because returning home is important to patients and their families as well as by policymakers because increased time in health institutions is inherently related to increased healthcare costs [3].

However, hometime also exhibits statistical properties which make its analysis difficult. First, hometime exhibits a highly non-normal bucket-shaped distribution with a spike at or near its lower and upper limits. Second, part of hometime’s non-normal distribution is made up of an inordinate excess of 0’s. Zero hometime can arise from two different scenarios: 1) the patient remained in a healthcare institution for the entire duration of follow up; or 2) the patient died before discharge from hospital and therefore could not accumulate any hometime. Typically, if the patient dies during the follow up window, any time spent at home before death is counted towards hometime [1–8, 10, 11]. However, in some studies, hometime has also been calculated such that patients who die at any point during the follow up window are assigned a hometime of 0 (even if they spent time at home during the observation window) [9]. Third, the lower and upper limits themselves cause difficulty in the analysis of hometime, as many traditional regression methods can result in predicted values of hometime that are outside of the range of possible values (e.g., predicting negative hometime or hometime beyond the upper limit of the observation window (i.e. predicting 100 days of hometime when the outcome of interest is 90-day hometime)).

In prior applied studies, a range of statistical methods have been used to analyze hometime; however, there have been no direct comparisons of different methodologies. Consequently, the optimal method for the analysis of hometime as an outcome is unknown. Additionally, there has been little use of methods from the machine learning literature for the analysis of hometime. In this study we aimed to compare the relative performance of different analytic strategies for predicting hometime. We performed these analyses in the context of stroke (both ischemic and hemorrhagic) as the index event causing hospitalization and the observation window to calculate hometime being 90 days.

### Description of the hometime modelling methods

We provide a brief description of different candidate approaches to model the effect of covariates on hometime. We will describe both traditional statistical and machine learning methods. Throughout the rest of this paper, we assume that the outcome is 90-day hometime, rather than hometime calculated using a different time period.

### Statistical models

#### Linear regression

Linear regression, estimated using ordinary least squares (OLS), has been used in the analysis of hometime in patients with sub-arachnoid hemorrhage [8]. An advantage of linear regression is that the model is additive, and the regression coefficients are easily interpreted as the change in mean hometime for a one unit increase in a given predictor variable. However, statistical inference using linear regression relies on the assumption that the error terms are normally distributed and have uniform variance. Hometime exhibits a highly non-normal distribution; consequently the distribution of error terms may have a non-normal distribution, which brings the inferences made from this model into question [12]. Additionally, the assumption of uniform variance likely does not hold true for hometime data. Finally, linear regression allows for predicted hometime to exceed the constraints on observed hometime, such as producing estimates < 0 or greater than the upper limit of the follow up window (90-days).

#### Ordinal logistic regression

The ordinal logistic regression (or proportional odds) model has been used to model hometime in patients with ischemic stroke [13]. An advantage of ordinal logistic regression specific to hometime is that it will not extrapolate beyond the range of possible outcome values, as it does not model the probability of having a value less than the minimum or greater than the maximum on the ordinal scale. However, this model makes the important assumption that the odds ratio assessing any effects of the exposure variable(s) on the outcome is invariant to the cut point used when the ordinal categories are dichotomized, which may not hold true for hometime [14, 15]. Another disadvantage of ordinal logistic regression is that it does not directly provide an estimated hometime for each individual in the sample; however, this can be overcome through calculation of the probability of each possible value of hometime for each individual, and then using these probabilities to determine the mean or expected hometime for each individual, conditional on their observed characteristics.

#### Poisson regression

The Poisson distribution is often used to model the distribution of hospital length of stay. Hometime can be thought of as similar to this, and as such could be modelled using Poisson regression. The advantage of using Poisson regression for hometime is that the fact that hometime is strictly non-negative is explicitly recognized. However, Poisson regression will allow for predicted values of hometime to exceed the upper limit of 90 days. Additionally, the use of the Poisson distribution relies on the assumption of equidispersion [16]; however, overdispersion is likely to be a problem with hometime data due to the spikes in hometime at 0 and near its upper limit of 90.

#### Negative binomial regression

Negative binomial regression has been used in a previous study to model hometime in patients with stroke [4]. Negative binomial regression is a generalization of Poisson regression which relaxes the assumption of equidispersion [16]. As with Poisson regression, the non-negative integer characteristics of hometime are explicitly recognized, but again it can result in predicted values of hometime that exceed the upper limit of 90 days.

#### Zero-inflated poisson regression and zero-inflated negative binomial regression

There are two reasons that a patient may have hometime = 0: the first being that they died in hospital and the second being that they remained alive but institutionalized until day 90. As such the hometime distribution suffers from excess zeros and zero-inflated methodologies such as zero-inflated Poisson regression or zero-inflated negative binomial regression may be appropriate for use. In these models it is assumed that the excess zeros are produced by a separate process from the rest of the count data and as such can be modelled separately [17]. Similar considerations made for using traditional Poisson or negative binomial regression need to be made here as well.

#### Hurdle regression

Hurdle models are another way of dealing with excess zeros and overdispersion which have been used before in the modelling of hometime in patients with stroke due to large vessel occlusion [11]. These are two-part models which specify separate processes for the zero counts and for the positive integer counts [17]. The premise is that a positive count occurs once a threshold (hurdle) is crossed, but if the threshold is not crossed the predicted count remains zero. Several different model types can be used for the zero process, including binomial, Poisson, negative binomial, or geometric distributions. For the positive integer counts Poisson, negative binomial, or geometric distributions can be used. The ability to use a variety of model types for both the zero count and positive integer count processes allows more flexibility than the zero inflated binomial and zero inflated negative binomial model families.

#### Cox proportional hazards regression

Proportional hazards models have not previously been used for modeling hometime. In using a proportional hazards model for hometime one is modelling “time to end of hometime” using the hazard function. One can then estimate the survival function for each patient and the area under the curve of the survival function can be used as an estimate of expected hometime. While this may seem like an unusual application of proportional hazards models, hazard models have some properties which may be useful in the analysis of hometime. Hometime’s complex distribution may lend itself better to semiparametric models, such as proportional hazards models [15]. Another advantage to using a proportional hazards model for hometime is that at day 90 the estimate survival function will be 0 for all patients. Consequently, the model will not produce estimates of expected hometime that exceed its theoretical lower and upper bounds.

### Machine learning methods

#### Ridge regression

Ridge regression (and lasso regression below) may be classified as both statistical and machine learning methods as they rely on a parametric model but use a data-driven approach to estimate the model coefficients. Unlike the least squares estimator used in linear regression which is designed to reduce the sum of squared residuals the ridge estimator is a shrinkage method which is designed at reducing the sum of squared residuals plus the L_2_ penalty which is made up of the sum of squared coefficients multiplied by $\lambda$ where $\lambda >0$. [18] This penalty introduces bias into the estimator; however, the bias results in lower variance. The size of the penalty is determined by $\lambda$ and the optimal $\lambda$ is chosen using cross validation. Ridge regression has not been used with hometime.

#### Lasso regression

The lasso estimator is similar to the ridge estimator, but it applies the L_1_ penalty to the estimator which is made up of $\lambda$ multiplied by the sum of the absolute values of the coefficients. Unlike ridge regression, the lasso estimator can shrink the coefficients to 0 whereas the in ridge regression the coefficients can only become asymptotically close to zero [18]. This means that lasso regression can also perform variable selection. Lasso regression has not been used with hometime data.

#### Support vector regression

Support vector regression is a variant of the support vector machine typically used for classification problems. In classification problems the goal is to find a hyperplane which optimally separates two classes of data. This hyperplane is a maximum margin separator, meaning that while minimizing error the hyperplane should also be at maximum distance from the different classes. This ensures that the support vector machine has good generalizability and is not prone to overfitting [18]. If perfect separation is not possible, slack variables are introduced to allow some error in misclassification (soft margin classifier). Support vector machines are generalized to the regression context by introducing an ε-insensitive region around the function (sometimes called the ε-tube). The value of ε determines the level of accuracy of the function and the number of support vectors used to construct the regression function. In support vector regression the aim is to find the flattest ε-tube that contains most of the training data while balancing model complexity and prediction error [19]. While support vector regression is a powerful prediction tool, it requires heavy computational time and storage requirements for large data sets.

#### Bagged regression trees

Bootstrapped aggregation (or bagging) was one of the earliest developed ensemble machine learning techniques, meaning its results are the combination of many models’ predictions [20]. In bagged regression trees, several (typically hundreds) of regression trees are generated from bootstrapped samples and predictions are averaged across the different regression trees. This aggregation can reduce prediction variance or noise in predictions. One downside to bagging is that trees can end up being very similar in structure, especially at the top of the tree, in a presence of strong predictors. This is known as correlation and when the bagged trees are highly correlated the reduction in variance desired by using bagging is often not achieved.

#### Random forests regression

Random forests regression has been used previously to model 90-day hometime in a population of patients with ischemic stroke or intracerebral hemorrhage [21]. Random forests are an extension of bagging where several hundred trees are grown from the same dataset and their results averaged. Like bagging, these trees are generated from bootstrapped samples of the full dataset. However, unlike bagging each time a split is considered only a random sample of predictors among the full set of predictors are chosen as candidates for the split. This both creates an improvement over bagging by decorrelating the trees and allows multicollinearity to be addressed as not all predictors are considered at each split [22]. The predictions for each observation from each tree are averaged to obtain the final predicted values. Random forests allow for complex interaction structures to be captured. However, as with other ensemble-based methods, they are considered a “black box” machine learning method, for which no interpretable regression coefficients are produced. This means that the direct interpretation of each variables impact on the outcome cannot be described without the use of additional measures such as calculating partial dependence.

#### Generalized boosting machines

Boosting is another ensemble machine learning technique in which multiple weak models are combined into a single strong model. Boosting begins with a series of weak learners which are simple algorithms with relatively high error rates. Unlike bagging or random forests, the individual models in the ensemble are not trained in parallel but rather are trained sequentially and each new model focuses on subjects for whom the previous model performed poorly. This allows for a focus on observations whose outcomes are difficult to predict with the goal of improving prediction for these subjects. Several different methodologies can be used within this algorithm including regression methods, Poisson models, and Cox proportional hazards models among others [23].

## Methods

### Cohort identification and data collection

The cohort of patients used in this study has been previously described [21]. In brief, all patients with a diagnosis of stroke (ischemic or intracerebral hemorrhage) admitted to an acute care hospital in Ontario between April 1, 2010 and December 31, 2017 were identified using the Canadian Institute for Health Information (CIHI) Discharge Abstract Database (DAD) using ICD 10 codes I61, I63, and I64. Exclusion criteria included non-residents of Ontario, those < 18 or > 105 years of age, those with stroke occurring in-hospital, patients with history of prior stroke, and patients in long-term care at baseline. Through data linkage, several covariates relevant to the prediction of long term outcomes after stoke were collected, including: age, sex, arrival by ambulance, stroke type, history of atrial fibrillation, diabetes, hypertension, myocardial infarction, treatment with thrombolysis, stroke unit care, frailty (measured using the Hospital Frailty Risk Score) [24], stroke severity (measured using the Passive Surveillance Stroke seVerity Indicator (PaSSV)) [25], rural vs. urban home location, and quintile of median neighbourhood income. Patients with missing data were excluded from these analyses.

Ninety-day hometime was calculated using data linkage of several administrative data sources spanning from acute to long term care. Data linkage occurred through unique encoded identifiers at ICES; these datasets have been linked and validated extensively for research purposes [26]. Ninety-day hometime was calculated as 90 minus the sum of length(s) of stay in any care setting. For patients who did not survive to day 90, the hometime calculation was censored at the date of death. Patients who died during the index admission had a hometime of 0 days by definition.

### Statistical methods

The study cohort was randomly split into a derivation sample (containing 2/3 of the patients) and a validation sample (containing the remaining 1/3 of the patients). All models were fit using the derivation sample. For all methods, full models were fit using all covariates and variable selection was not performed. For machine learning models the following parameters were used: for bootstrap aggregated regression trees 10,000 trees were grown. For random forests regression a random forest of 500 trees was grown using p/3 candidate predictors at each split (where p = total number of predictors), minimum node size was 5, and no restrictions on tree depth or number of terminal nodes were imposed. For support vector regression, epsilon regression with ε = 0.1 was used. For generalized boosting machines, two different parameter sets were used: one using the Gaussian distribution with an interaction depth of 2 and the second using the Poisson distribution with an interaction depth of 15 (several interaction depths were tested and those producing the best results in the derivation sample were chosen for use with the test sample). For lasso and ridge regression lambda values of 0.03 and 1.59 were used, respectively (chosen via tenfold cross validation in the derivation sample).

### Generating predicted hometime and evaluating predictive accuracy

The resultant fitted models were applied to the validation dataset. Thus, a predicted or expected hometime was obtained from each model for each subject in the validation sample. For a given prediction model, let ${\widehat{Y}}_{k}$ denotes the predicted hometime for the *k*th patient and ${Y}_{k}$ denotes the observed hometime for the *k*th patient. Model accuracy was determined by calculating the root mean square error (RMSE), mean absolute error (MAE), and bias in predicted hometime. These values were defined as follows:

$$RMSE= \sqrt{\frac{1}{n}\sum _{k}{\left({\widehat{Y}}_{k}-{Y}_{k}\right)}^{2}}$$

$$MAE= \frac{1}{n}\sum _{k}\left|{\widehat{Y}}_{k}-{Y}_{k}\right|$$

$$Bias= \frac{1}{n}\sum _{k}{\widehat{Y}}_{k}-\frac{1}{n}\sum _{k}{Y}_{k}$$

Model calibration was assessed using calibration plots and calibration slopes as outlined by Archer et al. [27] Calibration plots were generated by plotting actual hometime against predicted hometime values. The calibration slope $\left({\lambda }_{\mathrm{c}\mathrm{a}\mathrm{l}}\right)$ is derived from the calibration model which is fitted as follows:

$${Y}_{i}={\alpha }_{\mathrm{c}\mathrm{a}\mathrm{l}}+{\lambda }_{\mathrm{c}\mathrm{a}\mathrm{l}}\left({\widehat{Y}}_{k}\right)+{e}_{\mathrm{c}\mathrm{a}\mathrm{l}i}$$

Additionally, it was documented if the model constrained predicted values to the range of possible values for 90-day hometime values (from 0 to 90 inclusive).

The marginal effects of each continuous co-variate on the expected 90-day hometime were illustrated using partial dependence plots. These plots show how predicted values partially depend on the values of one or more co-variates. These graphs provide a method of model interpretation which plots the change in average predicted outcome value as a covariate is varied over its marginal distribution [28]. They do not reveal the inner workings of the model, but rather reveal how the model behaves as a result of changing inputs. One-way partial dependence plots were generated for each covariate. All analyses were performed using Stata13 and R v3.3.0.

### Ethics and data availability statement

This study was approved by the Sunnybrook Health Sciences Centre Research Ethics Board. The use of data in this project was authorized under Sect. 45 of Ontario’s Personal Health Information Protection Act. The first author had full access to all the data in the study and takes responsibility for its integrity and the data analysis. The data sets used for this study were held securely in a linked, de-identified form and analyzed at ICES. While data sharing agreements prohibit ICES from making the data set publicly available, access may be granted to those who meet pre-specified criteria for confidential access, available at www.ices.on.ca/DAS.

## Results

### Patient characteristics

**Fig. 1:**
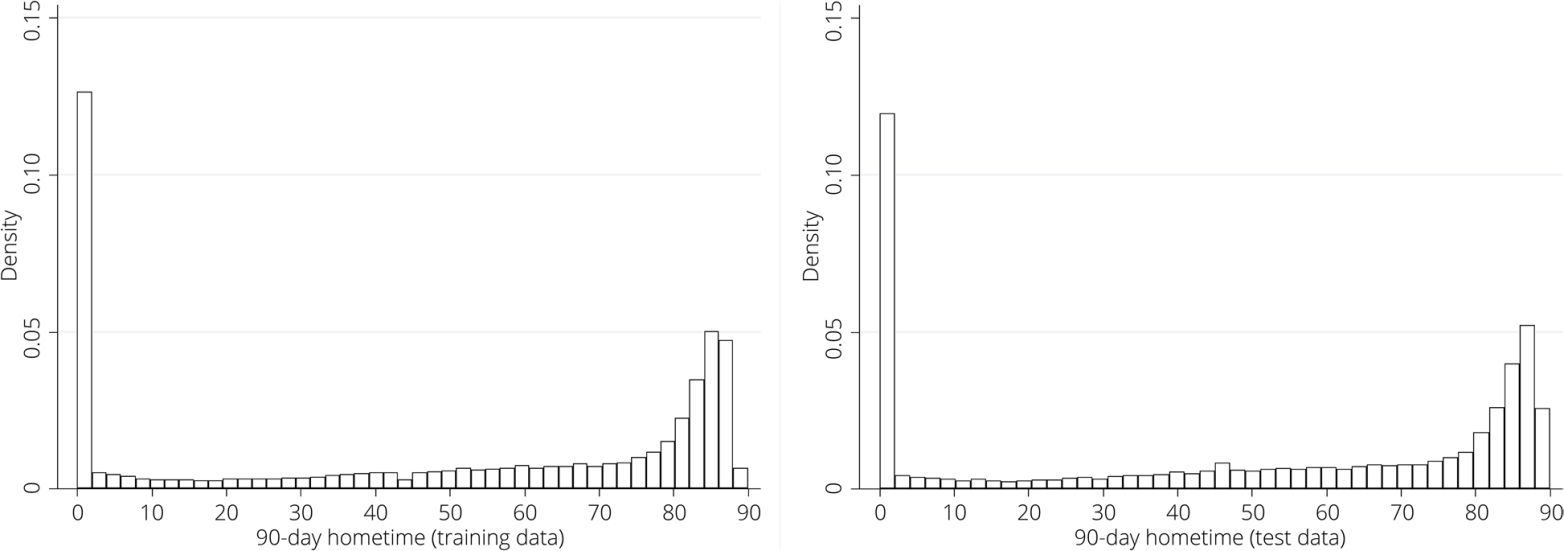
Distribution of 90-day hometime across training and test cohorts

We identified 75,475 patients. Baseline characteristics are described in Table 1. The median 90-day hometime across the cohort was 59 days (Q1: 2, Q3: 83) and at day-90 68.54% of patients were home and 17.49% of patients had died (Table 1). After the random split 49,402 observations were assigned to the derivation dataset and 26,073 to the validation dataset. The distribution of 90-day hometime in the derivation and validation datasets is shown in Fig. 1.

**Table 1:** Baseline characteristics and outcomes of study cohort

Characteristic	Study Cohort (n = 75,475)
Female (%)	47.44
Median Age (Q1, Q3) – years	75 (64, 84)
Arrived by Ambulance (%)	71.19
Stroke Type (%)	
Intra-cerebral Hemorrhage	12.87
Ischemic Stroke	87.12
Diabetes (%)	36.61
Atrial Fibrillation (%)	14.18
Hypertension (%)	82.76
Myocardial Infarction (%)	9.19
Neighbourhood Income Quintile (%)	
Quintile 1 (lowest)	23.60
Quintile 2	21.99
Quintile 3	19.70
Quintile 4	17.75
Quintile 5 (highest)	16.96
Home Location (%)	
Rural	12.40
Urban	87.60
Median Frailty Score^a^ (Q1, Q3)	4.2 (0.8, 9.1)
Median PaSSV Score^b^ (Q1, Q3)	7.7 (6.5, 8.7)
Received Thrombolysis (%)	13.36
Received Stroke Unit Care (%)	56.01
Median 90-day hometime (Q1, Q3)	59 (2, 83)
90-day location (%)	
Acute Care	4.14
Rehabilitation	2.91
Long Term Care	6.91
Home	68.54
Death	17.49

*Q1* First quartile, *Q3* Third quartile, *PaSSV* Passive Surveillance Stroke seVerity indicator

^a^A continuous score ranging from 0 – 99 where scores < 5 indicate low risk of frailty, scores from 5 – 15 indicate intermediate risk of frailty, and scores > 15 indicate high risk of frailty [24]

^b^A continuous score where < 4 indicates severe stroke, 4 – 8 indicates moderate stroke severity, and > 8 indicates mild stroke severity [25]

### Comparison of predictive models

The ability of each model to predict hometime in the validation dataset is reported in Table 2. The generalized boosting machine using the Poisson distribution with interaction depth = 15 produced the lowest RMSE at 27.89. This was closely followed by random forests regression (28.32) and the generalized boosting machine using the Gaussian distribution with interaction depth = 2 (28.39). The maximum RMSE of 30.15 resulted from negative binomial regression.

**Table 2:** Comparison of accuracy and bias metrics for predictive models used

Model	Root Mean Square Error	Mean Absolute Error	Bias	Minimum Predicted Value	Maximum Predicted Value	Calibration Slope
*Statistical Methods*						
Linear Regression	28.82	24.13	-0.26	-53.74	103.37	1.00
Ordinal Logistic Regression	28.64	23.96	-0.38	0.23	84.03	1.04
Poisson Regression	29.02	24.50	-0.25	2.90	144.98	0.95
Negative Binomial Regression	30.15	25.15	0.75	2.47	189.83	0.77
Zero Inflated Poisson Regression	28.47	23.68	-0.31	0.17	95.59	1.04
Zero Inflated Negative Binomial Regression	28.53	23.74	-0.31	0.18	97.46	1.03
Cox Proportional Hazards Model	29.29	25.62	-1.64	0.00	77.00	1.33
Hurdle Regression (negative binomial zero distribution, Poisson distribution)	28.47	23.65	-0.25	0.50	95.99	1.02
*Machine Learning Methods*						
Random Forests Regression	28.32	23.08	-0.40	0.04	85.83	0.98
Bagged Regression Trees	29.48	24.98	-0.25	18.20	73.29	1.06
Support Vector Regression	29.18	21.55	2.08	-17.91	91.99	0.74
Generalized Boosting Machine (Gaussian Distribution, Interaction Depth = 2)	28.39	23.89	-0.30	3.23	78.72	1.11
Generalized Boosting Machine (Poisson Distribution, Interaction Depth = 15)	27.89	22.81	-0.35	3.49	83.39	1.01
Lasso Regression	28.82	24.14	-0.26	-53.45	103.21	1.00
Ridge Regression	28.83	24.25	-0.27	-50.06	101.93	1.03

^*^a plausible minimum predicted value is ≥ 0, a plausible maximum predicted value is ≤ 90

The model with the lowest MAE was support vector regression (21.55, Table 2). Similar to RMSE, the generalized boosting machine using the Poisson distribution with interaction depth = 15 and random forests regression also produced low MAE (22.81 and 23.08 respectively, Table 2). The highest MAE (25.62) was produced by the Cox proportional hazards model.

Overall, bias was low across all models (Table 2). Bagged regression trees, Poisson regression, and hurdle regression produced the lowest bias of -0.25 days. With the exception of negative binomial regression and support vector regression all models underpredicted mean hometime. Negative binomial regression and support vector regression overpredicted hometime by relatively small amounts (0.75 and 2.08 days respectively).

The calibration slopes ranged from 0.74 to 1.33 across all models (Table 2). There was not a substantial difference in the range of slopes between the statistical and machine learning models. Support vector regression and negative binomial regression had the lowest calibration slopes (0.74 and 0.77 respectively), indicating that some of their predictions were too extreme. The Cox proportional hazards model and the generalized boosting machine using the Gaussian distribution (1.33 and 1.11 respectively) indicating that the range of predictions from these models may be too narrow. All other models produced calibration slopes near 1, with lasso and linear regression having calibration slopes of exactly 1. Calibration plots for all models are available in the supplemental materials.

Linear regression, lasso regression, ridge regression, and support vector regression produced implausible negative minimum values for hometime (-53.74, -53.45, -50.06, and -17.1 respectively); all other models produced minimum values of hometime which were plausible (i.e., greater than or equal to 0) (Table 2). Six of the models produced maximum values of hometime which were plausible (less than or equal to 90); these were bagged regression trees (73.29), Cox proportional hazards model (77.00), generalized boosting machine using the Gaussian distribution and interaction depth = 2 (78.72), generalized boosting machine using the Poisson distribution and interaction depth = 15 (83.39), ordinal logistic regression (84.03), and random forest regression (85.83).

**Fig. 2:**
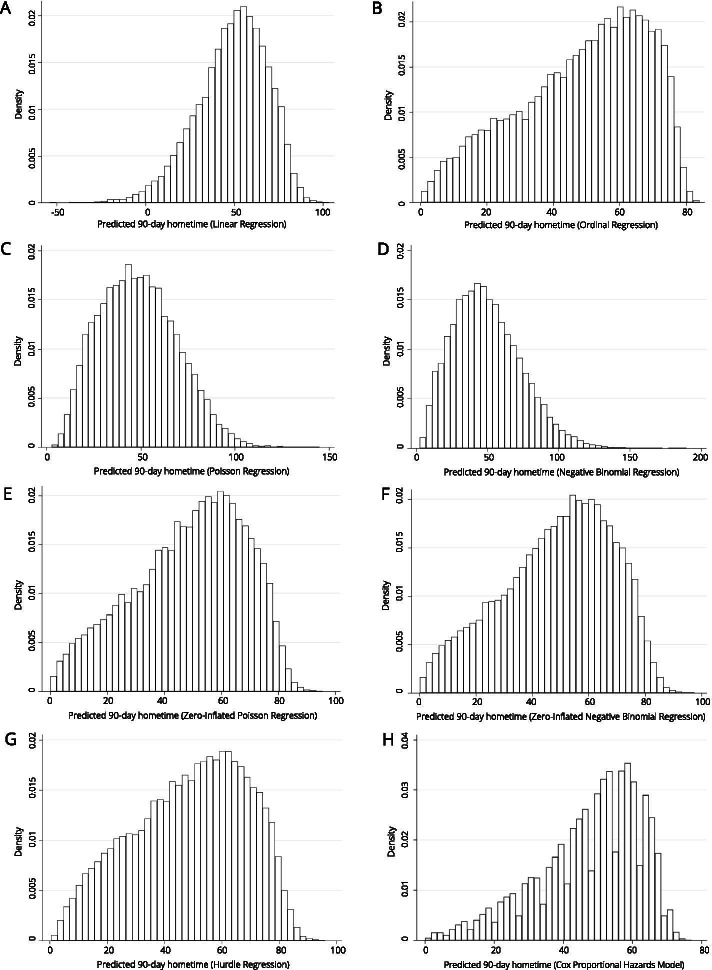
Distribution of predicted 90-day hometime across the test data set using eight different statistical models with 15 clinically relevant covariates (**A** Linear regression; **B** Ordinal logistic regression; **C** Poisson regression; **D** Negative binomial regression; **E** Zero-inflated Poisson regression; **F** Zero-inflated negative binomial regression; **G** Hurdle regression (negative binomial zero distribution, Poisson count distribution); **H** Cox proportional hazards model)

**Fig. 3:**
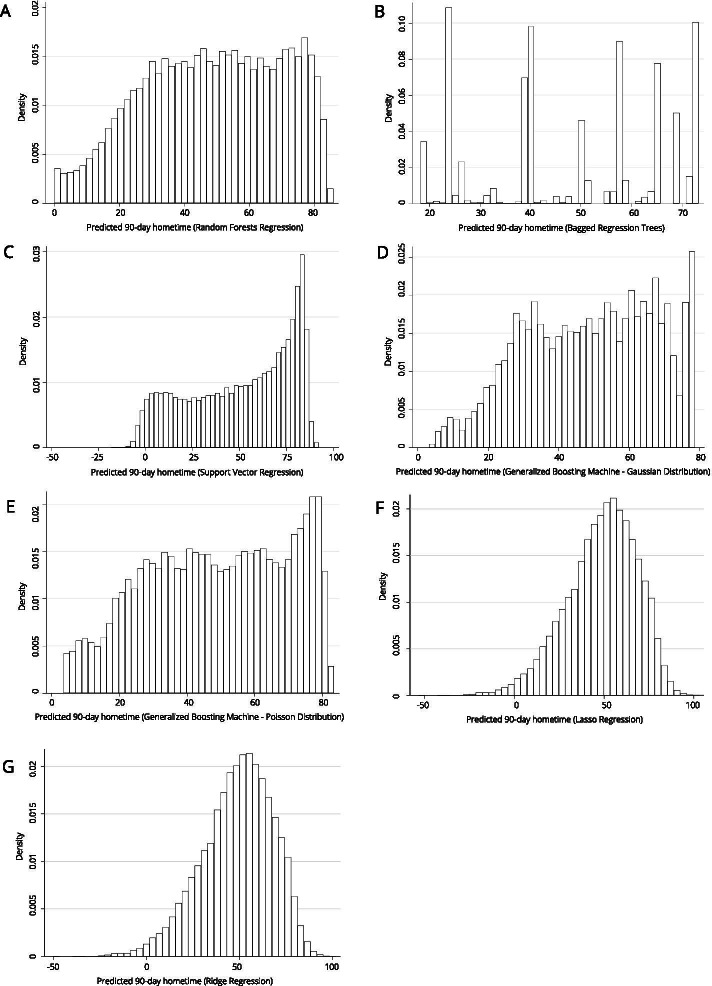
Distribution of predicted 90-day hometime across the test data set using seven different machine learning models with 15 clinically relevant covariates (**A** Random forests regression; **B** Bagged regression trees; **C** Support vector regression; **D** Generalized boosting machine (Gaussian distribution, interaction depth = 2); **E** Generalized boosting machine (Poisson distribution, interaction depth = 15)); **F** Lasso regression; **G** Ridge regression

The distribution of predicted hometime values for each model are displayed in Figs. 2 and 3. Many of the models result in a unimodal left skewed distribution of predicted values. Exceptions were Poisson regression and negative binomial regression, which both produced unimodal right skewed distributions (Fig. 2) and bagged regression trees which produced a multimodal distribution (Fig. 3). Random forests regression and both generalized boosting machines resulted in distributions which were relatively flat compared to those produced by the different generalized linear models which exhibited obvious peaks. While support vector regression did produce a spike in values near 90; none of the other distributions exhibited the spikes normally seen at or near the lower and upper limits of hometime.

### Marginal effects of covariates on the prediction of hometime

**Fig. 4:**
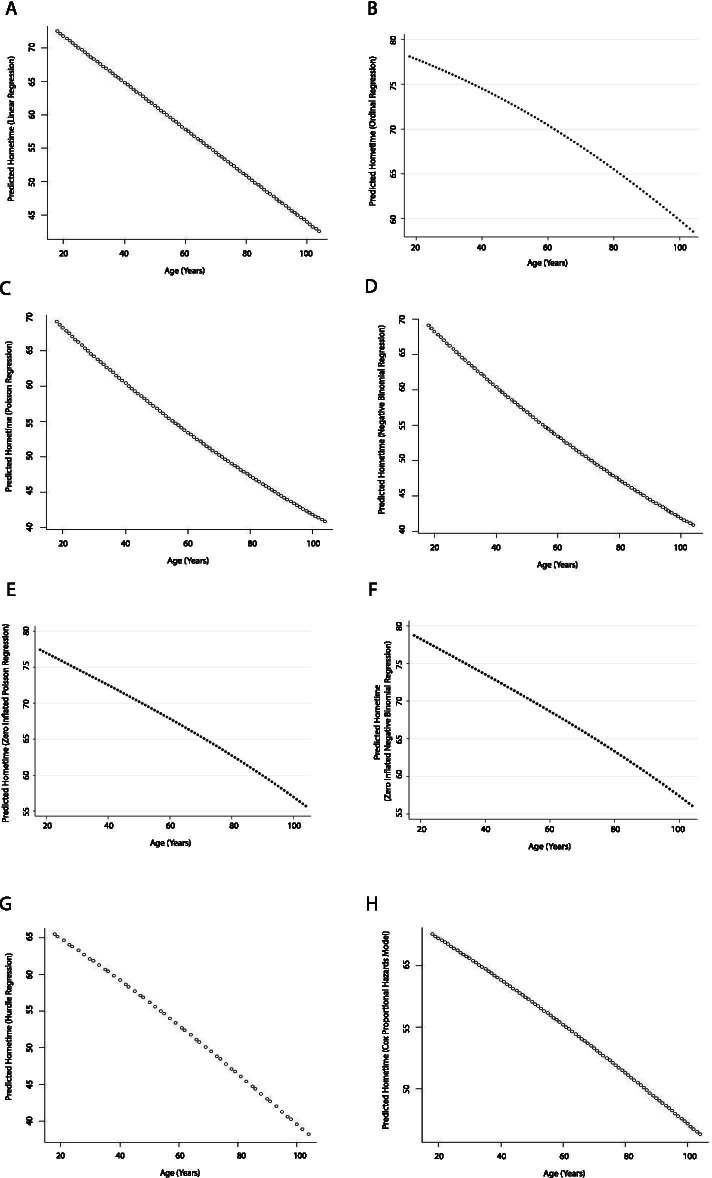
Partial dependence plots depicting the relationship between age and predicted 90-day hometime across the test data set using eight different statistical models (**A** Linear regression; **B** Ordinal logistic regression; **C** Poisson regression; **D** Negative binomial regression; **E** Zero-inflated Poisson regression; **F** Zero-inflated negative binomial regression; **G** Hurdle regression (negative binomial zero distribution, Poisson count distribution); **H** Cox proportional hazards model)

**Fig. 5:**
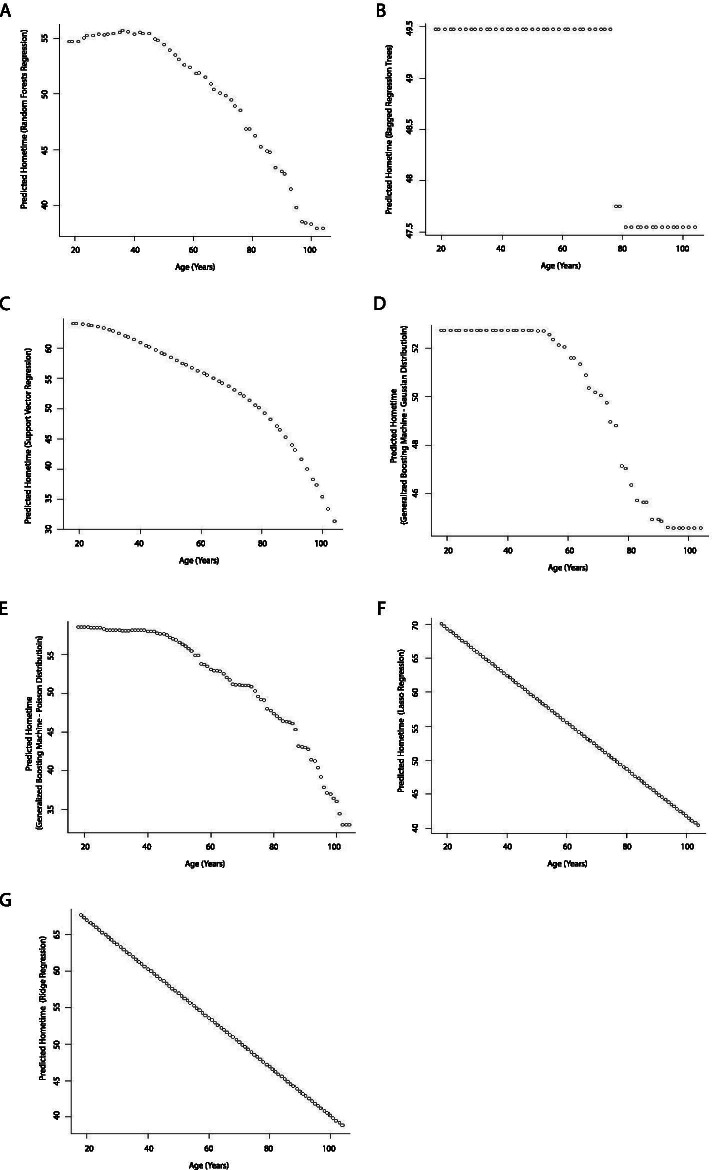
Partial dependence plots depicting the relationship between age and predicted 90-day hometime across the test data set using seven different machine learning models. (**A** Random forests regression; **B** Bagged regression trees; **C** Support vector regression; **D** Generalized boosting machine (Gaussian distribution, interaction depth = 2); **E** Generalized boosting machine (Poisson distribution, interaction depth = 15)); **F** Lasso regression; **G** Ridge regression

Age had an inverse relationship with hometime in all the models, which is consistent with the clinical observation that older patients have longer length of stay in health institutions or are more likely to die soon after the stroke, and therefore have less hometime than younger ones. The nature of this relationship varied with model type. As expected, the conventional statistical models as well as lasso and ridge regression showed linear relationships between age and hometime (Figs. 4 and 5). However, the other machine learning models all showed non-linear relationships (Fig. 5). The bagged regression tree analysis resulted in a step function whereas the other non-linear relationships showed hometime as high and relatively stable at younger ages and then rapidly dropped as age increased, the point at which the decline began varied between age 30 and 60 depending on the model used.

**Fig. 6:**
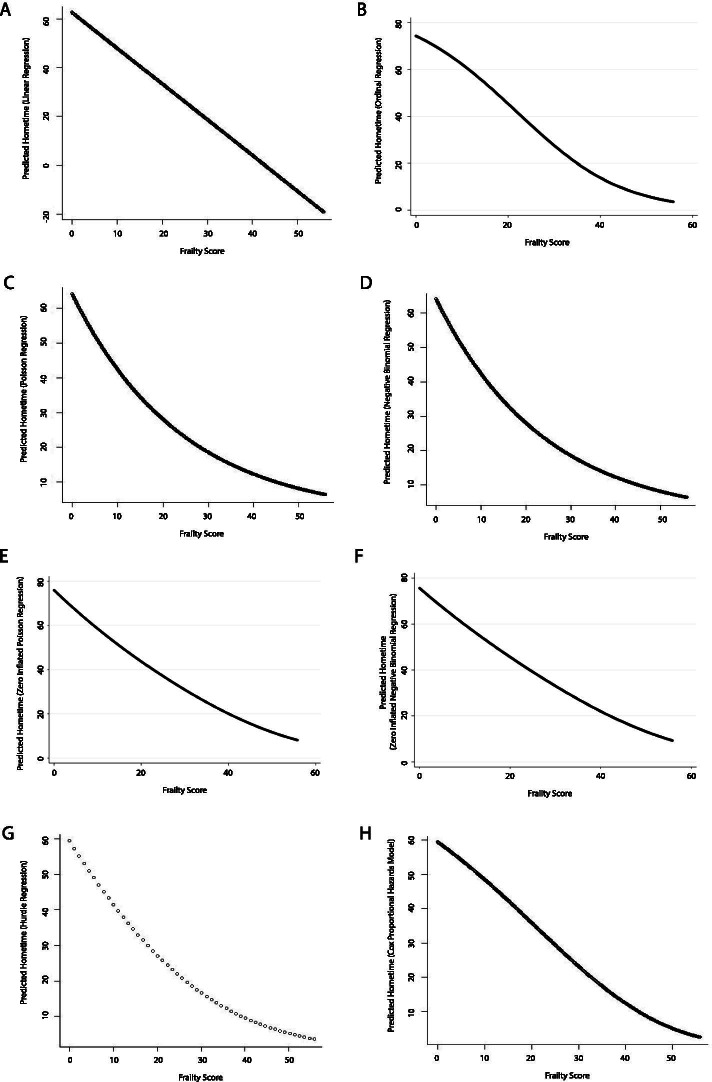
Partial dependence plots depicting the relationship between frailty score and predicted 90-day hometime across the test data set using eight different statistical models (**A** Linear regression; **B** Ordinal logistic regression; **C** Poisson regression; **D** Negative binomial regression; **E** Zero-inflated Poisson regression; **F** Zero-inflated negative binomial regression; **G** Hurdle regression (negative binomial zero distribution, Poisson count distribution); **H** Cox proportional hazards model)

**Fig. 7:**
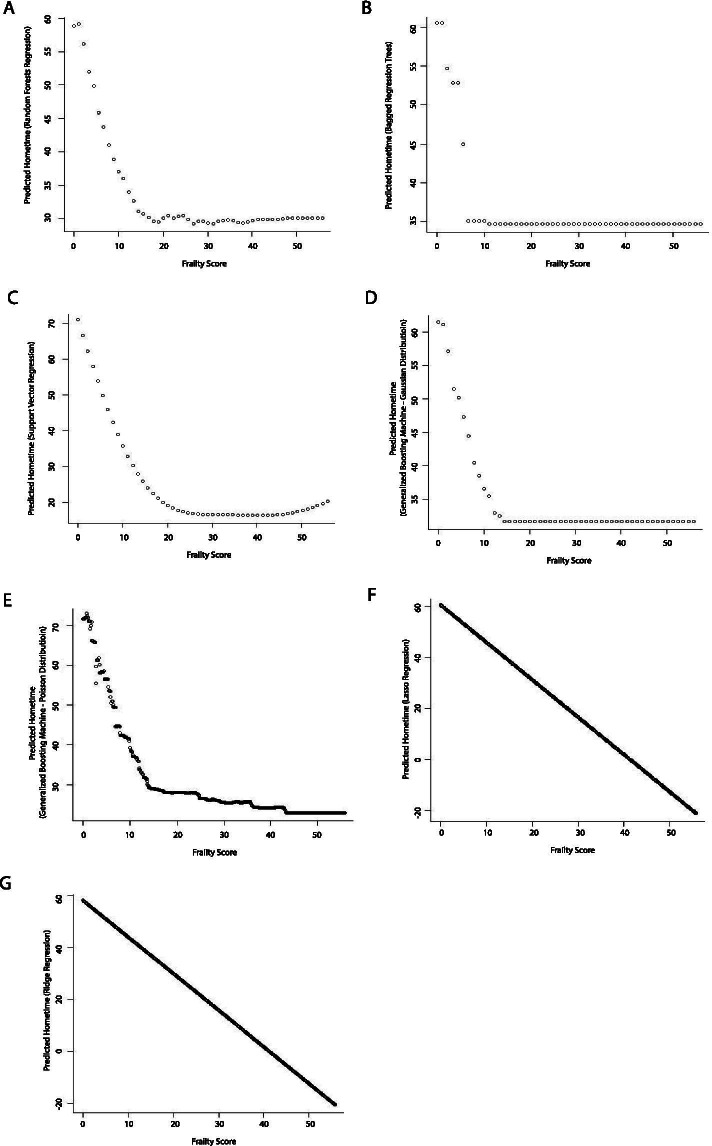
Partial dependence plots depicting the relationship between age and predicted 90-day hometime across the test data set using seven different machine learning models. (**A** Random forests regression; **B** Bagged regression trees; **C** Support vector regression; **D** Generalized boosting machine (Gaussian distribution, interaction depth = 2); **E** Generalized boosting machine (Poisson distribution, interaction depth = 15)); **F** Lasso regression; **G** Ridge regression

Frailty score also exhibited an inverse relationship with hometime. Again, the nature of this relationship varied with model type with those assuming linear relationships showing linear relationships (Figs. 6 and 7) and the other machine learning models showing non-linear relationships (Fig. 7). All machine learning models aside from ridge and lasso regression showed a steep drop in hometime as frailty score increased followed by relatively constant low hometime among higher frailty scores. The point at which hometime became relatively constant ranged between frailty scores of 5 to 15 depending on model used.

**Fig. 8:**
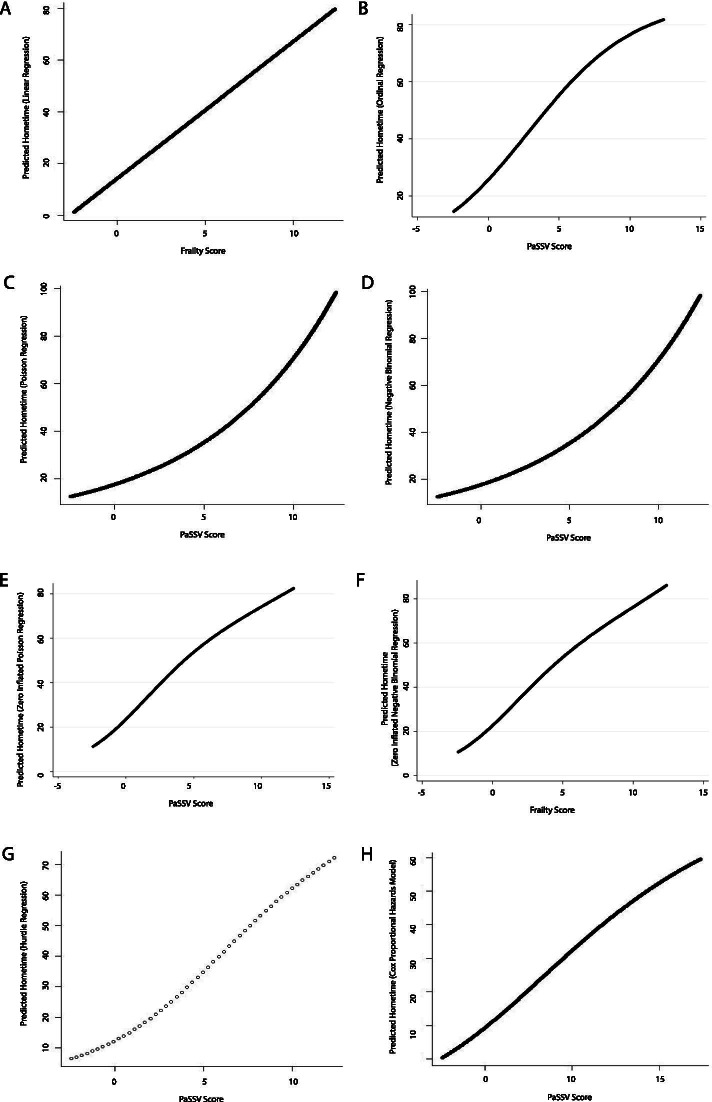
Partial dependence plots depicting the relationship between stroke severity (measured using the PaSSV score) and predicted 90-day hometime across the test data set using eight different statistical models (**A** Linear regression; **B** Ordinal logistic regression; **C** Poisson regression; **D** Negative binomial regression; **E** Zero-inflated Poisson regression; **F** Zero-inflated negative binomial regression; **G** Hurdle regression (negative binomial zero distribution, Poisson count distribution); **H** Cox proportional hazards model)

**Fig. 9:**
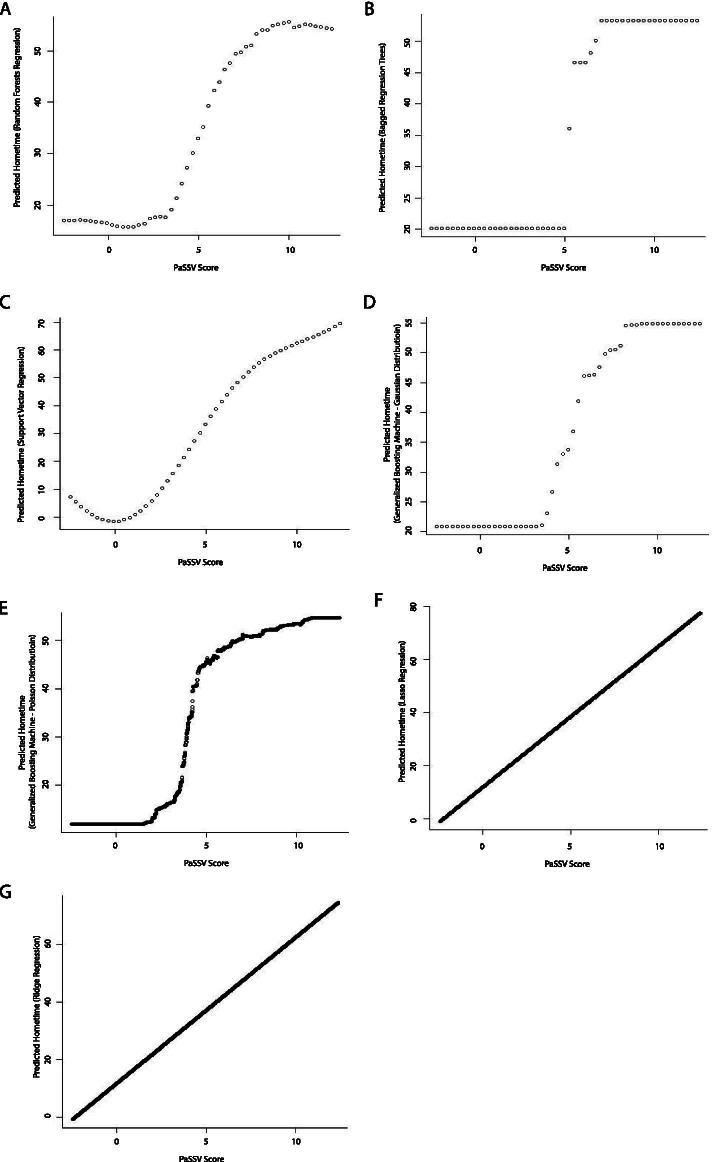
Partial dependence plots depicting the relationship between stroke severity (measured using the PaSSV score) and predicted 90-day hometime across the test data set using seven different machine learning models. (**A** Random forests regression; **B** Bagged regression trees; **C** Support vector regression; **D** Generalized boosting machine (Gaussian distribution, interaction depth = 2); **E** Generalized boosting machine (Poisson distribution, interaction depth = 15)); **F** Lasso regression; **G** Ridge regression

A direct relationship was observed between PaSSV score and hometime. Again, this relationship varied by model type, with most machine learning models displaying variations on an S-shaped relationship between PaSSV score and hometime where at low PaSSV scores hometime was low and relatively constant, hometime then rapidly increased through mid-range PaSSV scores and then again was high and relatively constant through higher PaSSV scores (Figs. 8 and 9). The one exception to this pattern was the support vector regression model which displayed a slightly different pattern whereby hometime did not flatten at higher values of PaSSV score and a small U-shaped relationship was seen at lower PaSSV scores.

## Discussion

We evaluated 15 models from the statistical and machine learning literature for the prediction of 90-day hometime in a cohort of 75,475 patients with stroke. Overall, there was not one model which clearly outperformed the others in terms of accuracy, bias, and range of predicted values.

Across all models the variability in RMSE and MAE was relatively low, spanning 27.89 to 30.15 and 21.55 to 25.62 respectively (Table 2). For both of these metrics, the machine learning models resulted in the lowest error; specifically, both generalized boosting machines and random forests regression had the lowest RMSE and support vector regression along with the generalized boosting machine (Poisson distribution) and random forests regression had the lowest MAE. However, not all the machine learning models outperformed the statistical models in this respect; bagged regression trees, which had the worst performance of the machine learning models, was outperformed by several of the statistical models. When evaluating bias this same trend of machine learning models resulting in the best performance was not observed. The models with the lowest bias were hurdle regression, Poisson regression, and bagged regression trees, which all underpredicted hometime by a mean of 0.25 days (Table 2). The largest bias resulted from support vector regression which overpredicted hometime by a mean of 2.08 days. There was no trend differentiating machine learning from statistical models in terms of calibration with most models being well calibrated (Table 2).

In terms of constraining the predicted values to those which are plausible (between 0 and 90 inclusive) the machine learning models outperformed the statistical models. All machine learning models, with the exception of support vector regression, lasso regression, and ridge regression, resulted in predicted values of hometime which were plausible. All of the statistical models, with the exception of linear regression, produced minimum predicted values which were plausible but only ordinal logistic regression and the Cox proportional hazards model produced maximum values which were plausible (Table 2).

Although many of the models performed reasonably well in terms of accuracy and bias, when comparing the distribution of predicted hometime to actual hometime, none of the models were able to capture the bucket-shaped distribution with spikes at 0 and near 90. Patients with these extreme values of hometime (0-hometime or very high hometime) were systematically under-represented in the distributions of predicted hometime, especially those with 0-hometime. As extreme values of hometime were poorly predicted across a wide range of different model types, we hypothesize that there may be factors strongly associated with either very low or very high hometime which were not captured in this study. Part of the difficultly may be that the 0-hometime group is not homogeneous. There are two different ways to arrive at 0-hometime: 1) the patient does not survive their initial stroke admission and thus never has the chance to accumulate any hometime, and 2) the patient remains institutionalized for the duration of the 90-days following their stroke. The characteristics of patients who die early and those who survived without the ability to return home are likely different. Interestingly, all models also systematically under predicted hometime values for patients with high hometime. Unlike 0-hometime, high hometime only has one interpretation, that the patient was sufficiently well for early discharge to home. It is plausible that some factors which could be associated with going home quickly (high hometime) may also be related to prolonged institutionalization (0-hometime). This includes factors like marital status, living situation, lifestyle factors, social support, and indicators of quality of care all of which are not readily available in administrative data. Future modelling studies of hometime using prospectively collected data may seek to include these types of variables.

We also explored the relationship between certain covariates of interest and hometime across the different model types using partial dependence plots. The machine learning models allowed for more flexibility in displaying non-linear relationships between continuous covariates and hometime. These complex non-linear relationships are likely more representative of what is seen in clinical practice. While these non-linear relationships could have also been captured using the conventional models, (ex. through the use of restricted cubic splines), we elected to use a simple implementation of these methods to reflect what is often done in practice. Put another way, the machine learning models allow the user to identify these complex non-linear relationships between covariates and hometime even if they aren’t specifically looking for them. However, the machine learning models come with the disadvantage that they do not readily produce regression coefficients which allow one to build equations for the prediction of hometime based on different patient level inputs.

## Conclusions

Hometime can be modelled with reasonable overall accuracy and low bias by many different model types. Machine learning models, especially the generalized boosting machine utilizing the Poisson distribution and random forests regression, exhibited the highest accuracy and least bias. However, no model was able to reproduce the bucket shaped hometime distribution with spikes at 0 and near 90, and future work will be needed to determine whether this is due to unmeasured variables which are associated with very high or very low hometime, and whether other analytic strategies are needed to address this.

## Supplementary Information

**Additional file 1:** **Figure S1**. Calibration plots displaying actual 90-day hometime plotted against predicted hometime across the test data set using eight different statistical models with 15 clinically relevant covariates. **Figure S2**. Calibration plots displaying actual 90-day hometime plotted against predicted hometime across the test data set using seven different machine learning models with 15 clinically relevant covariates.

## Data Availability

The data sets used for this study were held securely in a linked, de-identified form and analyzed at ICES. While data sharing agreements prohibit ICES from making the data set publicly available, access may be granted to those who meet pre-specified criteria for confidential access, available at www.ices.on.ca/DAS.

## Abbreviations

OLS: Ordinary least squares
CIHI: Canadian Institutes for Health Research
DAD: Discharge Abstract Database
PaSSV: Passive Surveillance Stroke seVerity Indicator
RMSE: Root mean square error
MAE: Mean absolute error
