# Supplementary material for: Comparing regression modeling strategies for predicting hometime
Source: BMC Med Res Methodol. 2021 Jul 7;21:138. doi: 10.1186/s12874-021-01331-9 (PMC8261957; doi:10.1186/s12874-021-01331-9)
Supplement: Supplementary file 1 — Additional file 1: Figure S1. Calibration plots displaying actual 90-day hometime plotted against predicted hometime across the test data set using eight different statistical models with 15 clinically relevant covariates. Figure S2. Calibration plots displaying actual 90-day hometime plotted against predicted hometime across the test data set using seven different machine learning models with 15 clinically relevant covariates. [file 12874_2021_1331_MOESM1_ESM.docx]

**Supplemental Materials:** Comparing regression modeling strategies for predicting hometime

Jessalyn K. Holodinsky, PhD, Amy Y.X. Yu, MD MSc, Moira K. Kapral, MD MSc, Peter C. Austin, PhD

**Supplemental Figures:**

Figure S1. Calibration plots displaying actual 90-day hometime plotted against predicted hometime across the test data set using eight different statistical models with 15 clinically relevant covariates

Figure S2. Calibration plots displaying actual 90-day hometime plotted against predicted hometime across the test data set using seven different machine learning models with 15 clinically relevant covariates

**Figure S1**. Calibration plots displaying actual 90-day hometime plotted against predicted hometime across the test data set using eight different statistical models with 15 clinically relevant covariates (A: linear regression; B: ordinal logistic regression; C: Poisson regression; D: negative binomial regression; E: zero-inflated Poisson regression; F: zero-inflated negative binomial regression; G: hurdle regression (negative binomial zero distribution, Poisson count distribution); H: Cox proportional hazards model)

**Figure S2.** Calibration plots displaying actual 90-day hometime plotted against predicted hometime across the test data set using seven different machine learning models with 15 clinically relevant covariates (A: random forests regression; B: bagged regression trees; C: support vector regression; D: generalized boosting machine (Gaussian distribution, interaction depth = 2); E: generalized boosting machine (Poisson distribution, interaction depth = 15)); F: lasso regression; G: ridge regression
